# Maternal overweight/obesity and yoghurt supplementation from early pregnancy to postpartum augments infant gut microbiota

**DOI:** 10.3389/fnut.2026.1733803

**Published:** 2026-02-26

**Authors:** Longlong Jia, Junying Zhao, Yanpin Liu, Yan Liu, Qian Liu, Bin Liu, Xianping Li, Guanghui Li, Ziyi Zhang, Minghui Zhang, Weicang Qiao, Huimin Liu, Lijun Chen

**Affiliations:** 1 College of Food Science and Engineering, Jilin Agricultural University, Changchun, Jilin, China; 2 National Engineering Research Center of Dairy Health for Maternal and Child, Beijing Sanyuan Foods Co. Ltd., Beijing, China; 3 Beijing Engineering Research Center of Dairy, Beijing Technical Innovation Center of Human Milk Research, Beijing Sanyuan Foods Co. Ltd., Beijing, China; 4 Key Laboratory of Dairy Science, Ministry of Education, Food Science College, Northeast Agricultural University, Harbin, China; 5 Division of Endocrinology and Metabolism, Department of Obstetrics, Beijing Obstetrics and Gynecology Hospital, Capital Medical University, Beijing, China

**Keywords:** gut microbiota, infant weight, overweight/obesity, pre-pregnancy, yoghurt

## Abstract

**Background:**

Maternal overweight/obesity during pregnancy heightens the risk of overweight/obesity in their offspring, and probiotic interventions during pregnancy may prevent excessive weight gain and enhance the abundance of beneficial gut microbiota. This study examined the effect of probiotic-rich yoghurt supplementation in overweight or obese women on infant weight and gut microbiota.

**Methods:**

The intervention group (YC) comprised 90 infants born to mothers with a pre-pregnancy body mass index (BMI) ≥ 25 kg/m^2^ who were provided with yoghurt from early pregnancy to 3 years postpartum. The control groups comprised 70 infants born to mothers with normal weight (NC) and 66 infants born to mothers with a BMI ≥ 25 kg/m^2^ (CC).

**Results:**

Infant weight was significantly higher in the YC group than in the NC group at 42 days and 3 months. The Shannon index of the YC group was higher than that of the NC group at 0–6 months. Enterotype compositions in the YC and CC groups differed from those of the NC group during 0–6 months. In the NC group, the relative abundance of *Akkermansia* and *Serratia* remained stable, that of *Blautia* was initially stable but then increased, whereas that of *Lactobacillus* decreased over time. In the CC and YC groups, the abundance of these genera increased and then decreased. Infant weight and the relative abundance of *Veillonella*, *Fusicatenibacter*, and *Akkermansia* showed a positive correlation.

**Conclusion:**

Maternal overweight/obesity affects subsequent infant weight and gut microbiota development, and maternal yoghurt intervention may alter the relative abundance of infant gut microbiota.

## 1 Introduction

Maternal health and lifestyle influence the short- and long-term development of infant gut microbiota and health (1). Pre-pregnancy overweight/obesity is associated with an elevated infant birth weight (2, 3) and may increase the risk of obesity in one-year-old infants by threefold (4). Postpartum maternal weight is positively correlated with infant birth length, weight, and head circumference percentiles (5). Maternal weight may affect infant weight by altering breast milk composition. Infant birth weight and adiponectin content in breast milk show a significant positive correlation, which is influenced by maternal body mass index (BMI) (6). Another study found that pregnant women who are overweight (7) or with pre- and postnatal stress (8) can reduce the alpha diversity of their child’s gut microbiota.

The gut microbiota is a microbial ecological community that plays an important role in nutrient absorption (9). Maternal diet affects the development of the infant gut microbiota. Maternal vitamin D levels lead to significant changes in the diversity and species composition of gut microbiota in 1- and 6-month-old infants (10), and maternal fruit and vegetable intake during pregnancy is associated with differences in the composition of infant gut microbiota at 2 months (11). In contrast, high-fat diets are associated with a reduced gut diversity, and fibre intake may enhance microbial diversity (12). Exposure to probiotics during pregnancy may influence the establishment of an infant’s gut microecology (13). Probiotic supplementation in pregnant women who are overweight/obese was found to increase the richness and diversity of maternal gut microbiota, which may slow down weight gain during pregnancy, and this effect is related to the duration of intervention (14). Some of the probiotics ingested by the mother may colonize the infant’s gut and thereby alter the gut microbiota composition (15, 16). Maternal intake of probiotics could increase the abundance of some gut microbiota of the infant and reduce infant body weight (17). Bovine milk contains high-quality protein, phospholipids, oligosaccharides, vitamins, and other nutrients and is a good carrier of both prebiotics and probiotics. Yoghurt contains healthy microbial metabolites (postbiotics) and is a recommended food for maternal diets. However, clinical evidence of its potential benefits for the short- and long-term health of infants remains lacking.

In this study, we conducted a continuous yoghurt intervention from early pregnancy to 3 years postpartum in women with overweight/obesity to determine the impact of maternal yoghurt intervention on their children’s growth and development and on the formation of the infant gut microbiota. Two control groups (no intervention) included infants born to mothers who were either overweight/obese or of normal weight.

## 2 Materials and methods

### 2.1 Participants

Pregnant women were recruited based on the following inclusion criteria: age 18–35; singleton pregnancy; 8–12 weeks pregnant; Beijing resident (>5 years); willing and able to complete the questionnaire; and no pre-pregnancy hypertension, diabetes, or infectious diseases. Exclusion criteria were as follows: multiple pregnancies; administration of antibiotics and probiotics or prebiotics in the month preceding the study; chain smoker or alcoholic; psychiatric disorders; prior bariatric surgery; and lactose intolerance or milk protein allergy.

### 2.2 Experimental design

Based on the China maternal and infant nutrition health birth cohort study (MINC), pregnant women with overweight/obesity (pre-pregnancy BMI ≥ 25 kg/m^2^) were randomly assigned via computerised randomisation to either the yoghurt intervention (YC) group or the overweight/obese control (CC) group. From early pregnancy through approximately 4 years postpartum, the YC group were provided and instructed to consume 200 mL daily of yoghurt enriched with probiotics and prebiotics (see Supplementary Table 1 for details). Yoghurt was delivered to participants daily via cold chain transport. Their yoghurt intake was tracked and recorded throughout the study period to ensure that participants consumed the specified quantity at the designated times. Additionally, pregnant women of normal weight (pre-pregnancy BMI < 23.9 kg/m^2^) were enrolled via random sampling to form the normal-weight control group (NC group). All women were prescribed diets and exercise during pregnancy by a professional dietitian based on the maternal dietary guidelines developed by the Chinese Maternal and Child Health Association and all mothers were instructed that the provided yogurt was for their exclusive consumption and not to be given to their babies.

Follow-up monitoring was conducted at days 5 and 42, and at months 3, 6, 12, 18, 24, 30, and 36 postpartum. Infant weight and height were measured by nurses on days 5 and 42, and by mothers at 3–36 months, using a standardised method and calibrated equipment. At each follow-up time point, completed dietary questionnaires for mothers and infants were collected to examine probiotic and antibiotic intake.

### 2.3 Sample collection

At each follow-up visit, infant faecal samples were collected using collection tubes containing stabilising solution, placed at −20 °C for storage after collection, and transferred to −80 °C within 24 h.

### 2.4 DNA extraction and sequencing

DNA was extracted from faecal samples using a DP336 extraction kit (Tiangen Biotech (Beijing) Co., Ltd., Beijing, China). The amount of DNA obtained from the samples was ≥3 μg, with a concentration ≥50 ng/μL and purity OD_260/280_ = 0.7–1.9. Each polymerase chain reaction (PCR) mixture contained 15 μL Phusion High-Fidelity PCR Master Mix (New England Biolabs, Inc., Ipswich, MA, USA), 0.2 μM primers, and 10 ng genomic DNA template. The amplification protocol involved initial denaturation at 98 °C for 1 min, followed by 30 cycles of denaturation at 98 °C for 10 s, annealing at 50 °C for 30 s, an extension at 72 °C for 30 s, and a final extension at 72 °C for 5 min. PCR products were purified using magnetic beads; equal volumes were pooled based on their concentrations, mixed thoroughly, and resolved by agarose gel electrophoresis. The constructed library was quantified using a Qubit fluorometer and quantitative PCR. After quantification, PE250 up-sequencing was performed using a NovaSeq 6000 sequencing system.

### 2.5 Data processing methods

Sequencing data were processed using the QIIME2 platform, and the spliced Raw Tags strictly filtered using fastp software (v.0.23.1) to obtain high-quality Tags (Clean Tags) (18). They were then compared with bacterial species annotation databases (19) to detect chimeric sequences, yielding a set of effective data (Effective Tags) (20). Noise reduction was performed using the DADA2 module in the QIIME2 software, and species annotation performed on the aggregated data based on the Silva annotation database (v.138.2).

The resulting data were analysed using R (v.4.4.0). The baseline characteristics of volunteers and infant weight were analysed using one-way analysis of variance (ANOVA), followed by Tukey’s post-hoc test for multiple comparisons. Alpha and beta diversity analyses were performed using the vegan package (21); all images in the text were plotted using the ggplot2 package (22). To determine differences among groups, ANOVA was used for normally distributed data; otherwise, the Wilcoxon signed-rank test was used. All samples were clustered into enterotypes using the partitioning around medoids clustering algorithm (23), the difference of enterotypes between different groups were compared using the chi-square test. Pearson correlation analysis was performed using the Hmisc package (24).

Infants of different sexes were assessed according to the weight percentile corresponding to their age. The method of assessing the clinical weight development grade of infants was based on the percentile evaluation method in the growth standard for children under 7 years of age in China (WS/T 423-2022) (25), as follows: weight percentile ≥ 97% was the upper grade; 75% ≤ weight percentile < 97% the upper-middle grade; 25% ≤ weight percentile < 75% the middle grade; 3% ≤ weight percentile < 25% the lower-middle grade, and weight percentile < 3% the lower grade. The differences of infant/child weight grade between the different groups were compared using the chi-square test. With false discovery rate correction for multiple comparisons applied to all resulting *p-*values (unadjusted) to yield adjusted *p*-values (*p_adj_*).

## 3 Results

### 3.1 Cohort description

This study enrolled 318 pregnant women, of whom 226 continued to participate in the project during the postpartum period. These 226 participants included 90 in the YC group, 66 in the CC group, and 70 in the NC group (Supplementary material 2). Birth mode and feeding method showed no significant differences among the three groups, all of which were full-term births (Table 1). No significant differences in pre-pregnancy weight or BMI were observed between the YC (75.7 ± 10 kg, 28.5 ± 3.22 kg/m^2^) and CC (72.8 ± 11.4 kg, 28 ± 4.11 kg/m^2^) groups, whereas these parameters were significantly lower in the NC group (57.8 ± 9.12 kg, *p_adj_* < 0.001; 21.7 ± 3.57 kg/m^2^, *p_adj_* < 0.001). Maternal age did not differ significantly between the YC group (32.5 ± 3.85) and the CC group (33.2 ± 3.86), but it was significantly higher in both groups compared with the NC group (30.0 ± 4.65, *p_adj_* < 0.05). Moreover, no significant differences in infant weight and length at birth were observed among the three groups (Table 1).

**Table 1:** Maternal and infant characteristics.

Group	YC (*n* = 90)	CC (*n* = 66)	NC (*n* = 70)	Adjusted *p-*value
Mother’s age	32.5 ± 3.85^b^	33.2 ± 3.86^b^	30 ± 4.65^a^	0.038
Birth weight (kg)	3.8 ± 0.986^a^	3.47 ± 1.02^a^	3.46 ± 0.406^a^	0.431
Birth length (cm)	51.8 ± 2.95^a^	53 ± 4.24^a^	50.5 ± 0.829^a^	0.131
Mother’s height (cm)	163 ± 5.48^a^	161 ± 5.33^a^	163 ± 4.09^a^	0.140
Pre-pregnancy weight (kg)	75.7 ± 10^b^	72.8 ± 11.4^b^	57.8 ± 9.12^a^	<0.001
Pre-pregnancy BMI	28.5 ± 3.22^b^	28 ± 4.11^b^	21.7 ± 3.57^a^	<0.001
Breast milk/formula	46/44	34/32	40/30	0.716
Vaginal delivery/Caesarean section	39/51	33/33	39/31	0.298

Row values with different subscript letters are significantly different.

### 3.2 Comparison of weight gain in infants

**Figure 1:**
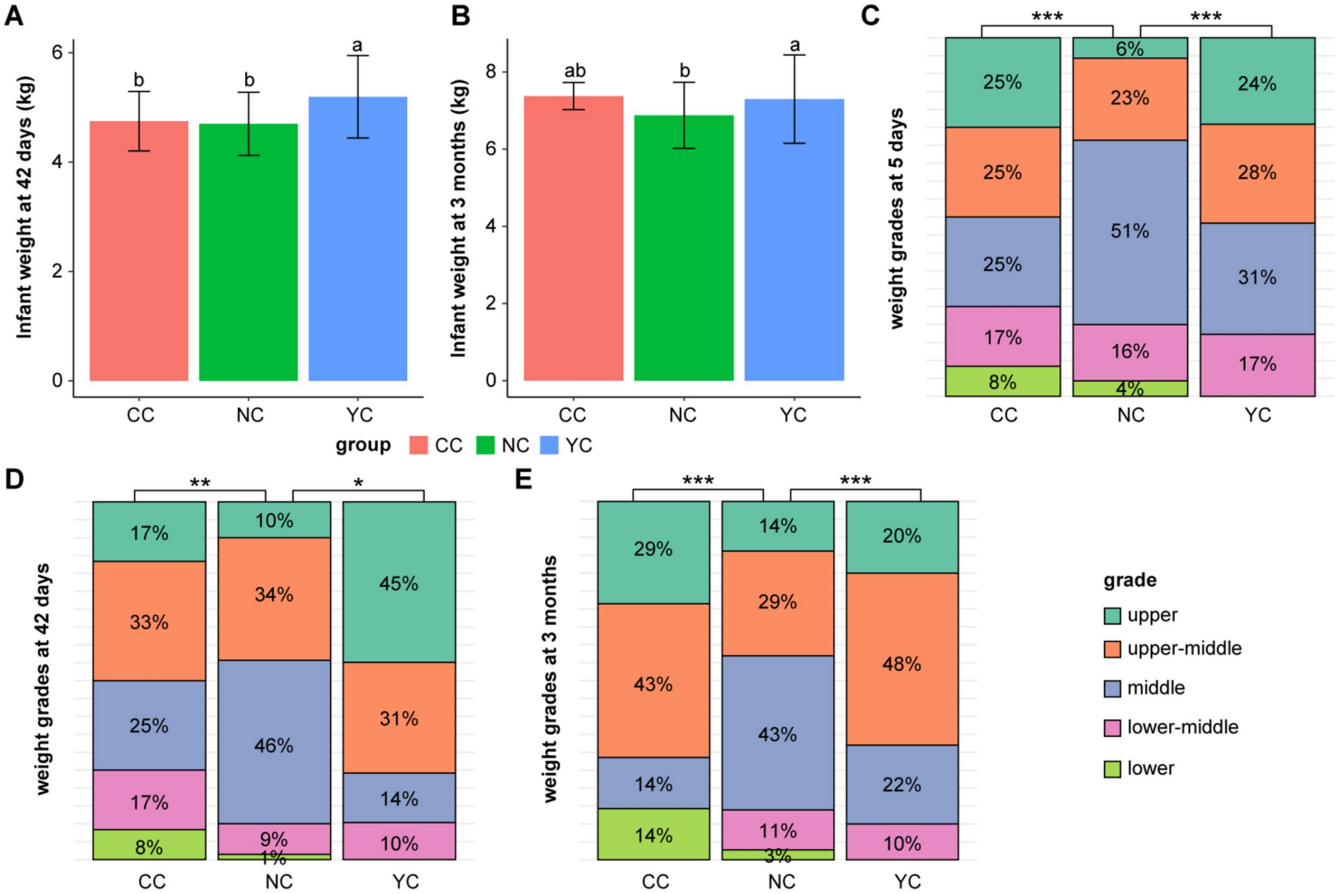
Infant weight development. **(A–B)** Comparison of the difference in weight at **(A)** 42 days and **(B)** 3 months among the three groups of infants, data are reported as the mean ± standard error of the mean; error bars indicate the SEM. **(C–E)** Comparison of the percentile and difference in weight grades at **(C)** 5 days, **(D)** 42 days, and **(E)** 3 months of age among the three groups of infants. Significant differences are indicated as follows: ***p_adj_ < 0.001, **p_adj_ < 0.01, *p_adj_ < 0.05.

At 42 days, infant weight in the YC group (5.22 ± 0.75 kg) was significantly higher than that in the NC (4.7 ± 0.57 kg; *p_adj_* < 0.001) and CC (4.64 ± 0.82 kg; *p_adj_ <* 0.05) groups, with no significant difference observed between the CC and NC groups (Figure 1A). At 3 months, infant weight in the YC group (7.33 ± 1 kg) was significantly higher than that in the NC group (6.88 ± 0.87 kg; *p_adj_ <* 0.05) (Figure 1B). At 6–36 months, children’s weight was higher in the YC and CC groups than in the NC group, but the difference was not significant. No significant differences were observed in the height and BMI of infants among the three groups (Supplementary Table 2).

According to the WS/T 423-2022 growth standard, infant weight is divided into five grades—lower, lower-middle, middle, upper-middle, and upper. At 5 days (*p_adj_* < 0.05), infant weight in the NC group was predominantly middle and upper-middle grade (74%), with a small proportion of upper grade (6%); 59% in the YC group was of middle and upper-middle grade, and 24% upper grade (Figure 1C). At 42 days (*p_adj_* < 0.001), 46 and 10% of infant weight in NC group was of middle and upper grade, respectively; 14 and 45% in the YC group was of middle and upper grade, respectively (Figure 1D). At 5 and 42 days, no significant difference was observed between the CC and the other two groups. At 3 months, no difference in weight grade was observed between the YC group and the NC and CC groups. However, a significant difference was noted between the CC and NC groups (*p_adj_* < 0.05), with infant weight in the CC group being 43% middle-upper grade and 29% upper grade, whereas 29% in the NC group was of upper-middle grade and 14% of upper grade (Figure 1E).

### 3.3 Comparison of gut microbiota diversity and enterotype in infants

**Figure 2:**
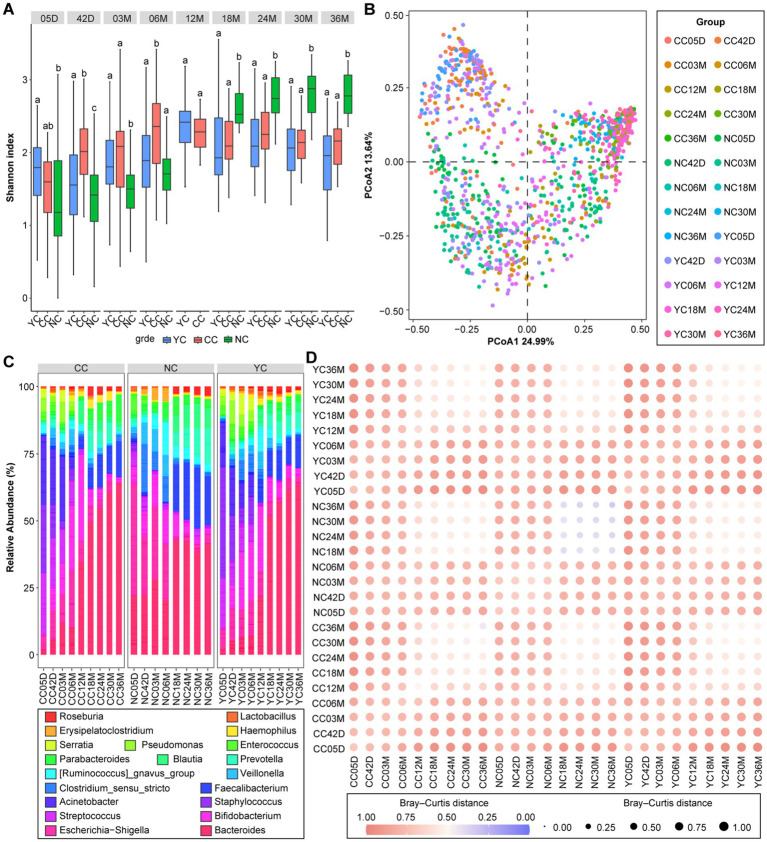
Characterisation of infant and children gut microbiota development. **(A)** Shannon index of infants and children in the three groups at all follow-up times. **(B)** Principal co-ordinates analysis (PCoA) of the three groups of infants and children at all follow-up times. **(C)** The 20 most abundant bacterial genera of the gut microbiota identified at different follow-up times in the three groups of infants and children. **(D)** Heatmap showing the similarity in infant gut microbiota based on the mean Bray–Curtis distances between the sample groups.

The alpha diversity (Shannon index) of gut microbiota in the three groups of infants progressively increased with age from 5 days to 36 months after birth (Figure 2A). At 5 days, the Shannon index was significantly higher in the YC group (1.76 ± 0.53) than in the NC group (1.38 ± 0.78; *p_adj_* < 0.001). At 42 days, it was significantly higher in the YC (1.68 ± 0.74) and CC (2.03 ± 0.53) groups than in the NC group (1.39 ± 0.47; *p_adj_* < 0.05). At 3 months, it was significantly higher in the YC (1.82 ± 0.52) and CC (1.96 ± 0.75) groups than in the NC group (1.43 ± 0.42; *p_adj_* < 0.001). At 6 months, it was significantly lower in the YC (1.86 ± 0.67) and NC (1.71 ± 0.46) groups than in the CC group (2.31 ± 0.53; *p_adj_* < 0.001). At 18, 24, 30, and 36 months, the Shannon index was significantly higher in the NC group than in the YC and CC groups (*p_adj_* < 0.001), with no significant differences observed between the YC and CC groups.

The Bray–Curtis distance indicated that infant gut microbiota were mainly influenced by age and groups (PERMANOVA *R*^2^ = 0.26, *p_adj_* < 0.001). Significant differences in the gut microbiota β-diversity were observed among the YC, CC, and NC groups at all follow-up times, with no significant differences noted between the YC and CC groups (Figure 2B). The top 20 most abundant bacterial genera of the gut microbiota in the three groups showed differences at different follow-up times (Figure 2C). The relative Bray–Curtis distances among the three groups during 0–18 months of age were greater than those among the corresponding three groups during 18–36 months of age (Figure 2D).

**Figure 3:**
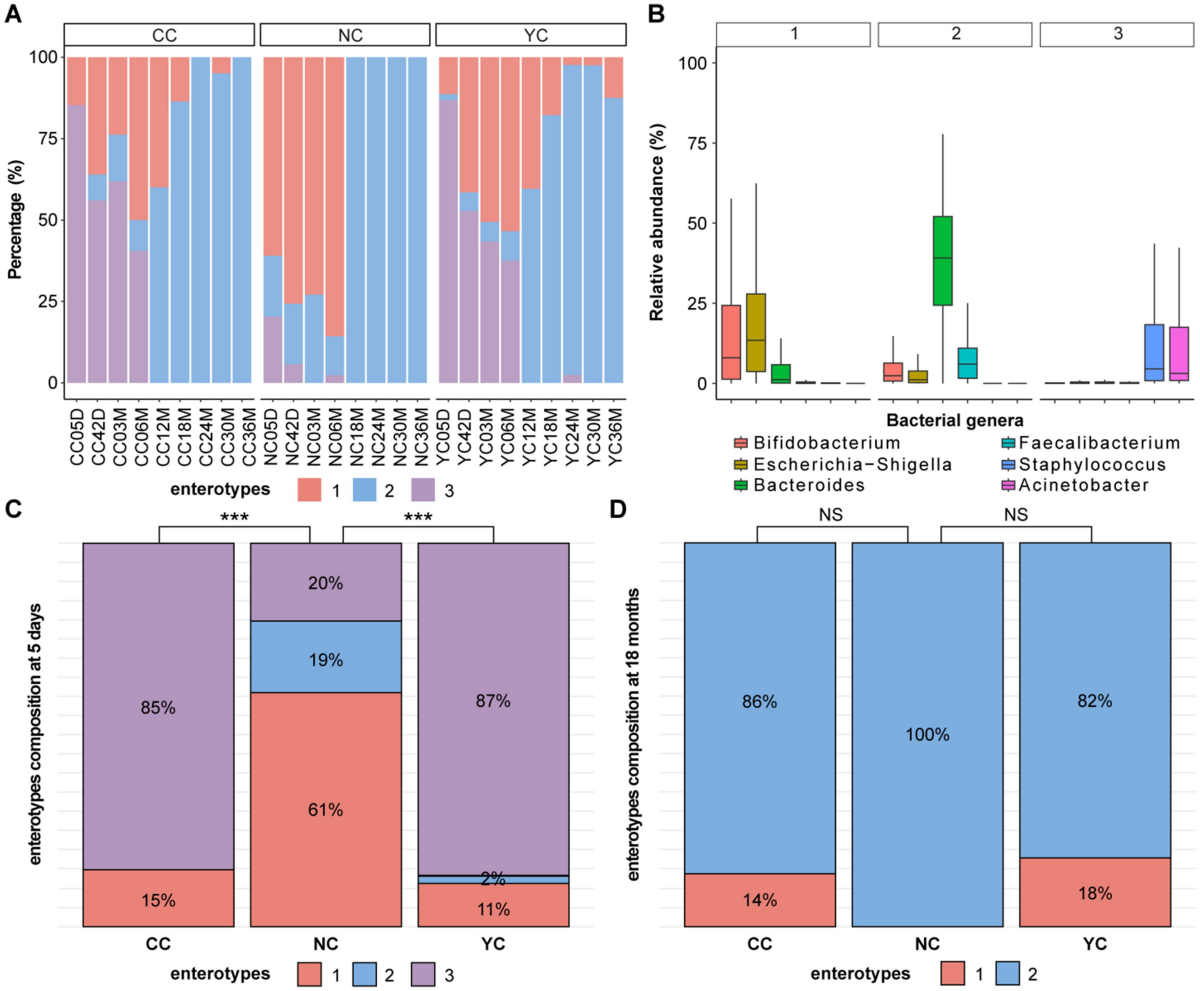
Analysis of enterotypes in the three groups of infants and children. **(A)** Composition of enterotypes at different follow-up times for the three groups of infants and children. **(B)** Three enterotypes represent the relative abundance of bacterial genera. **(C,D)** Differences in the composition of enterotypes among the three groups of children at **(C)** 5 days and **(D)** 18 months of age. Significant differences are indicated as follows: ***p_adj_ < 0.001, **p_adj_ < 0.01, *p_adj_ < 0.05, NS, not significant (*p_adj_* > 0.05).

The gut microbiota of infants aged 0–3 years can be classified into three enterotypes (Figure 3A), with *Escherichia-Shigella* and *Bifidobacterium* as the representative genera of enterotype 1; *Bacteroides* and *Faecalibacterium* as the representative genera of enterotype 2; and *Staphylococcus* and *Acinetobacter* as the representative genera of enterotype 3 (Figure 3B). From 5 days to 6 months, the enterotype composition differed significantly among the YC, CC, and NC groups (*p_adj_* < 0.05). For the YC and CC groups, enterotypes 1 and 3 predominated, which differed from the NC group, where enterotype 1 predominated (Figure 3C). No significant difference in the composition of enterotypes was observed among the three groups after 12 months, wherein enterotype 2 predominated in all groups (Figure 3D).

### 3.4 Comparison of gut microbiota pattern and abundance in infants

**Figure 4:**
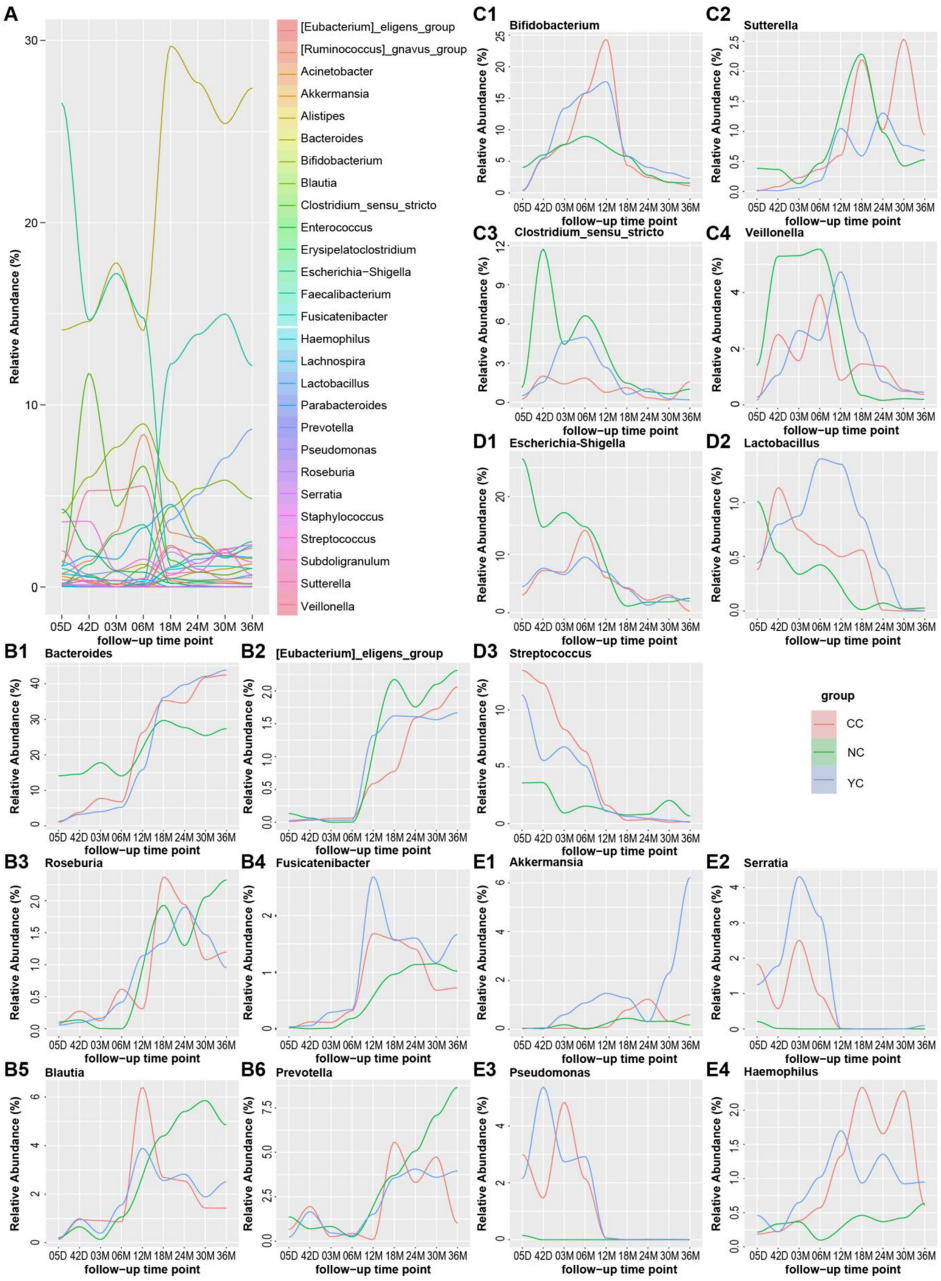
Changes in the relative abundance of gut microbiota in the three groups of children. **(A)** Changes in the relative abundance of infant gut microbiota in the NC group at different time points. **(B)** Changes in the abundance of gut microbiota that follow pattern 1: initially stable, followed by an increase over time in the three groups. **B1–B6** represent *Bacteroides*, [*Eubacterium*]*_eligens_group*, *Roseburia*, *Fusicatenibacter*, *Blautia*, and *Prevotella*, respectively. **(C)** Changes in the abundance of gut microbiota that follow pattern 2: increasing and then decreasing over time in the three groups. **C1–C4** represent *Bifidobacterium*, *Sutterella*, *Clostridium_sensu_stricto*, and *Veillonella*, respectively. **(D)** Changes in the abundance of gut microbiota that follow pattern 3: decreasing over time in the three groups. **D1–D3** represent *Escherichia-Shigella*, *Lactobacillus*, and *Streptococcus*, respectively. **(E)** Changes in the abundance of gut microbiota that follow pattern 4: stable over time in the three groups. **E1–E4** represent *Akkermansia*, *Serratia*, *Pseudomonas*, and *Haemophilus*.

Four patterns in relative abundance over time of gut bacterial genera were observed in the NC group during the 0–36 month period (Figure 4A): initially stable and then increasing (pattern 1); increasing and then decreasing (pattern 2); decreasing over time (pattern 3); and stable over time (pattern 4). Some genera showed similar changing patterns in the NC group as well as in the YC and CC groups, such as the relative abundance of *Bacteroides*, *Faecalibacterium*, [*Eubacterium*]_*eligens*_group, *Lachnospira*, *Prevotella*, and *Subdoligranulum*, all of which were stable during the first 6 months of life, with a gradual increase observed thereafter (Figure 4B, Supplementary Figure 1A). The abundance of *Bifidobacterium*, [*Ruminococcus*]_*gnavus*_group, *Veillonella*, *Sutterella*, *Clostridium_sensu_stricto*, *Erysipelatoclostridium*, *Parabacteroides*, and *Alistipes* increased before 12 months and decreased thereafter (Figure 4C, Supplementary Figure 1B). The abundance of *Enterococcus*, *Streptococcus*, and *Escherichia-Shigella* gradually decreased (Figure 4D, Supplementary Figure 1C), whereas that of *Acinetobacter* and *Staphylococcus* did not change significantly over time (Figure 4E, Supplementary Figure 1D). Others showed different changing patterns in the YC and CC groups than in the NC group. The relative abundance of *Roseburia*, *Fusicatenibacter*, and *Blautia* was stable in the NC group during the first 6 months and increased thereafter. However, in the CC and YC groups, it initially increased and then decreased over time (Figures 4B,B3–B5). The relative abundance of *Lactobacillus* decreased in the NC group, whereas it initially increased and then decreased over time in the CC and YC groups (Figures 4D,D2). The relative abundance of *Akkermansia*, *Serratia*, *Pseudomonas*, and *Haemophilus* decreased in the NC group over time but initially increased and then decreased in the CC and YC groups (Figures 4E,E1–E4).

**Figure 5:**
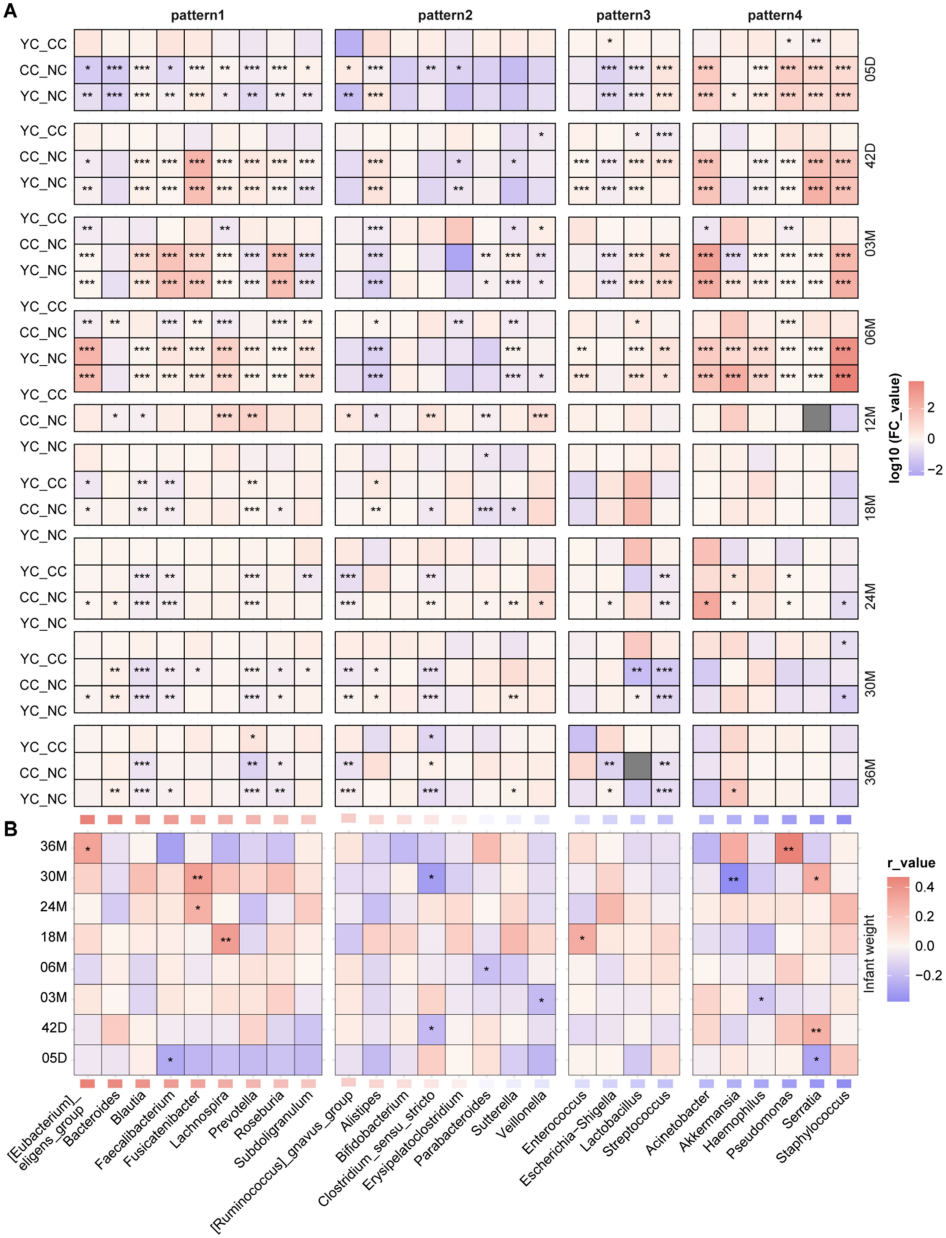
Differences in gut microbiota among the three groups of infants and children correlation with their weight. **(A)** Heatmaps of differences in gut microbiota over time across the three groups of children, with fold-change values calculated from YC/CC, YC/NC, and CC/NC comparisons. Significant differences are indicated as follows: ***p_adj_ < 0.001, **p_adj_ < 0.01, *p_adj_ < 0.05. **(B)** Heatmaps of the correlation between infant and child weight and gut microbiota abundance; significant differences are indicated as follows: ***p < 0.001, **p < 0.01, *p < 0.05.

Differences in the relative abundance of gut microbiota genera among the three groups were further analysed (Figure 5A). After 18 months, the relative abundance of *Blautia* was significantly lower in the YC and CC groups than in the NC group and was higher (but not significantly so) in the YC group than in the CC group. The relative abundance of *Prevotella* significantly differed among the YC, CC groups and the NC group, but no clear pattern was observed. From 5 days to 6 months, the relative abundance of *Fusicatenibacter*, *Pseudomonas*, and *Serratia* was significantly lower in the NC group than in the YC and CC groups, and the relative abundance of *Roseburia* was significantly higher in the NC group than in either the YC or CC groups. At 5 days and at 3 and 6 months, the relative abundance of *Akkermansia* was significantly higher in the YC group than in the NC group. Differences in the relative abundance of infant gut microbiota were also observed in the YC and CC groups. The relative abundance of *Lactobacillus* was significantly higher in the YC group than in the CC group at 6 months and was higher (but not significantly so) in the YC group than in the CC group at other follow-up times. *Pseudomonas* abundance was significantly lower in the YC group than in the CC group at 5 days and 3 months. At 3 months and 12 months, *Veillonella* abundance was significantly higher in the YC group than in the CC group.

Significantly negative and positive correlations were observed between infant weight and the relative abundance of *Serratia* at 5 (*r* = −0.29, *p* ≤ 0.01) and 42 days (*r* = 0.28, *p* < 0.01), respectively. A significantly negative correlation was observed between infant weight and the relative abundance of *Veillonella* (*r* = −0.21, *p* ≤ 0.01) at 3 months. At 30 months, infant weight showed a significantly positive correlation with the relative abundance of *Fusicatenibacter* (*r* = 0.36, *p* < 0.01) and *Serratia* (*r* = 0.3, *p* < 0.05) and a significantly negative correlation with the relative abundance of *Clostridium_sensu_stricto* (*r* = −0.32, *p* < 0.05) and *Akkermansia* (*r* = −0.37, *p* < 0.01) (Figure 5B).

## 4 Discussion

This study analysed the effect of a probiotic-rich yoghurt intervention from early pregnancy to 3 years postpartum in mothers with overweight/obesity on infant growth and infant gut microbiota. At 42 days and 3 months, we found that infant weights were significantly higher in the YC group than in the NC group. Moreover, when comparing the 5- and 42-day weight grades, the percentages of the middle, upper-middle, and upper weight grades in the YC and CC groups were higher than those in NC group infants, suggesting that infants of women who were overweight/obese during pregnancy are at an increased risk of obesity, which is consistent with the current results (26–29). Nutritional interventions, such as probiotics and prebiotics, are considered crucial for regulating the gut microbiota in obesity (30). Previous clinical trials have demonstrated that administering probiotics to mothers can mitigate excessive weight gain in infants during early infancy (31). In the present study, providing mothers with probiotic–prebiotic yoghurt did not significantly reduce their child’s weight but prevented excessive weight gain. This may be because the child’s weight is influenced far more by maternal weight than by the yoghurt intervention, suggesting that directly providing yoghurt to infants and children may be a more effective intervention for managing their weight.

The Shannon index of infants of women with overweight/obese was significantly higher than that of infants born to women of normal weight in the first 6 months, and significantly lower after 18 months, which is consistent with a literature report (32). Additionally, no significant differences in the Shannon index of the gut microbiota was observed between the YC and CC groups, except at 42 days and 6 months postpartum. This suggests that the diversity of the gut microbiota in children of overweight/obese women differs significantly from that in infants of women of normal weight, and that the yoghurt intervention did not have a sufficiently strong effect. The β-diversity analysis of the gut microbiota during the 0–36 month period revealed significant differences in composition between children of mothers with overweight/obesity compared to those of women who are of normal weight, but these differences gradually decreased, suggesting that the effects of maternal weight and yoghurt intervention on the child’s gut microbiota decreased over time.

In the present study, the infant and children’s gut microbiota was divided into three enterotypes, among which enterotype 1 (*Escherichia*-*Shigella* and *Bifidobacterium*) was similar to the infant enterosignatures (33); enterotype 2 (*Bacteroides* and *Faecalibacterium*) was similar to the adult enterotype (23); but enterotype 3, represented by *Streptococcus* and *Acinetobacter*, has not previously been reported. Before 1 year of age, the infants born to mothers with normal weight predominantly have enterotype 1, and those born to mothers with overweight/obese have a mixture of enterotypes 1 and 3. Previous studies observed slow weight gain in preterm infants with low *Streptococcus* abundance (34) and an association between *Acinetobacter* and rapid infant growth (35). *Streptococcus* and *Acinetobacter* dominated enterotypes 3 and may have facilitated more rapid infant weight gain, which may be one reason infants of overweight/obese pregnant women tend to be heavier. After 1 year of age, enterotype 2 became predominant in all three groups of children, suggesting that maternal overweight/obesity affects the development of the infant’s gut microbiota, but this effect diminishes over time. Yoghurt intervention in pregnant women with overweight/obesity had no significant effect on infant enterotype establishment.

We found that in the NC, YC, and CC groups, the relative abundance of some bacterial genera showed the same changing patterns, such as *Bacteroides, Faecalibacterium, Prevotella*, and *Bifidobacterium,* and some bacterial genera showed different changing patterns and obvious abundance differences, such as *Blautia*, whose abundance was different among the three groups after 18 months, in the order of NC > YC > CC. Intervening with probiotics during pregnancy can affect the gut microbiota of infants (36). In the present study, higher Lactobacillus abundance was observed in infants born to overweight/obese pregnant women, and a yoghurt intervention resulted in a higher *Lactobacillus* abundance at 6 months. *Lactobacillus* has been considered a candidate for obesity-resistant strains in previous studies (37, 38) and has also found to significantly reduce abdominal adiposity and inhibit obesity in obese mice (39, 40). Thus, lower levels of *Lactobacillus* may lead to obesity (41). We found a higher abundance of *Lactobacillus* in infants of the YC group, indicating that pregnant women with overweight/obesity who ingested yoghurt during pregnancy could increase the abundance of *Lactobacillus* in their infants, which may have beneficial effects on infant weight control. *Pseudomonas* might cause several diseases in infants (42), and in the first 6 months, children of women of normal weight had a significantly lower *Pseudomonas* abundance than those born to women with overweight/obesity. Infants born to women with overweight/obesity who received yoghurt intervention showed a lower *Pseudomonas* abundance. This finding provides preliminary evidence that maternal yoghurt intervention might be beneficial to the infant gut microbiota.

We also found that differences in the relative abundance of infant gut bacterial genera may be associated with infant body weight. *Roseburia* abundance and body weight were negatively but not significantly correlated, which was consistent with previous reports (43, 44). The children born to women of normal weight had a significantly higher *Roseburia* abundance at 5 days than those of women with overweight/obesity, which may explain the lower body weights of these infants. A negative correlation was observed between *Veillonella* abundance and infant weight at 3 months; *Veillonella* abundance was significantly higher in preterm infants with growth disorders than in healthy infants (45). Children born to mothers with overweight/obesity had a low *Veillonella* abundance at 3 months, which may have contributed to the higher weights of these infants observed at this period.

Consistent with previous reports (46–48), we observed positive correlation between *Fusicatenibacter* abundance and children body weight at 30 months, whereas *Akkermansia* exhibited negative association with infant weight at the same age. At 30 months, *Fusicatenibacter* abundance was significantly higher in children of the NC group than in those of the CC group, whereas children in the NC group had a lower *Akkermansia* abundance than those in the YC group. Children born to women with overweight/obesity often exhibit a higher weight, with NC group infants having high *Fusicatenibacter* and low *Akkermansia* abundances, which may explain the lack of differences observed in infant weights at this period.

Our study had certain limitations. Infant samples and clinical data for the NC group were not collected at 12 months due to unforeseen circumstances; additionally, some participants withdrew from the study after 12 months, resulting in a paucity of post-12-month clinical data.

## 5 Conclusion

This study assessed the effect of yoghurt supplementation in women with overweight/obesity from early pregnancy to 3 years postpartum and its effect on their children’s growth and gut microbiota development. At 0–6 months, body weight and the Shannon index of infants born to women with overweight/obesity were higher than those of infants born to women of normal weight. At 0–36 months, significant differences could be observed in gut microbiota composition and the abundance of genera, such as *Blautia*, *Lactobacillus*, *Veillonella*, *Fusicatenibacter*, and *Akkermansia*, between children of women with overweight/obesity and those of women of normal weight. Yoghurt supplementation in women with overweight/obesity during pregnancy augmented the gut microbiota, such as *Lactobacillus* and *Akkermansia*, in their children.

## Data Availability

The datasets presented in this study can be found in online repositories. The names of the repository/repositories and accession number(s) can be found in the article/Supplementary material.
