# Supplementary material for: Maternal overweight/obesity and yoghurt supplementation from early pregnancy to postpartum augments infant gut microbiota
Source: Front Nutr. 2026 Feb 26;13:1733803. doi: 10.3389/fnut.2026.1733803 (PMC12979164; doi:10.3389/fnut.2026.1733803)
Supplement: Supplementary file 1 [file Supplementary_file_2.doc]

**Flow Diagram**

**Enrollment**

**Allocation**

**Follow-Up**

**Analysis**

China maternal and infant nutrition health birth cohort study (n=1300)

Excluded (n=1034)

♦  Other areas (n=826)

♦  Other reasons (n=110)

Lost to follow-up (No samples provided) (n= 39 )

Allocated to Yogurt intervention OW/OB (n=140)

Randomized controlled(n=250)

Allocated to No-intervention OW/OB (n=110)

Allocated to normal weight(n=114 )

Analysed (n=90 )
♦ Excluded from analysis (Sample missing) (n=11 )

Lost to follow-up (No samples provided) (n= 38 )

Analysed (n=66 )
♦ Excluded from analysis (Sample missing) (n=6 )

Analysed (n=70 )
♦ Excluded from analysis (Sample missing) (n=12 )

Lost to follow-up (No samples provided) (n= 32 )

Random sampling(n=114)
