# Supplementary material for: Maternal overweight/obesity and yoghurt supplementation from early pregnancy to postpartum augments infant gut microbiota
Source: Front Nutr. 2026 Feb 26;13:1733803. doi: 10.3389/fnut.2026.1733803 (PMC12979164; doi:10.3389/fnut.2026.1733803)
Supplement: Supplementary file 2 [file Table_1.docx]

**Supplementary Table 1.** Yoghurt ingredients.

| Category | Name | Additive amount |
| --- | --- | --- |
| Raw materials | Raw milk |  |
|  | *Streptococcus* *thermophilus* |  |
|  | *Lactobacillus* *bulgaricus* |  |
|  | Steviol glycoside | 1–2% |
|  | Mogroside | 1–2% |
| Dietary fiber | Oligofructose | 6 g/100 g |
|  | Galacto-oligosaccharides |  |
|  | Polydextrose |  |
|  | Maltooligosaccharide |  |
|  | Xylo-oligosaccharide |  |
|  | Inulin |  |
| Multiple bacteria | *Lactobacillus* *acidophilus* | 1 × 10^8^ CFU/100 g |
|  | *Lactobacillus* *plantarum* |  |
|  | *Lactobacillus* *paracasei* |  |
|  | *Bifidobacterium* *breve* |  |
|  | *Bifidobacterium* *lactis* |  |
|  | *Bifidobacterium* *longum* |  |
|  | *Lactobacillus* *plantarum* | 1 × 10^8^ CFU/100 g |

CFU, colony-forming units
