# Supplementary material for: Maternal overweight/obesity and yoghurt supplementation from early pregnancy to postpartum augments infant gut microbiota
Source: Front Nutr. 2026 Feb 26;13:1733803. doi: 10.3389/fnut.2026.1733803 (PMC12979164; doi:10.3389/fnut.2026.1733803)
Supplement: Supplementary file 3 [file Table_2.docx]

**Supplementary Table 2.** Comparison of infant height and body mass index.

| Infant-clinical | Group | CC | NC | YC |
| --- | --- | --- | --- | --- |
| Height | 05 days | 50 ± 2.9^a^ | 50 ± 0.83^a^ | 50 ± 2.95^a^ |
| Height | 42 days | 55 ± 3.94^a^ | 56 ± 2.53^a^ | 56.5 ± 3.02^a^ |
| Height | 03 months | 64.1 ± 3.73^a^ | 63 ± 3.85^a^ | 64 ± 3.42^a^ |
| Height | 06 months | 70 ± 3.61^a^ | 69 ± 2.56^a^ | 70 ± 2.77^a^ |
| Height | 18 months | 84.8 ± 4.18^a^ | 82 ± 3.78^a^ | 84 ± 2.89^a^ |
| Height | 24 months | 90 ± 9.52^a^ | 89.5 ± 9.19^a^ | 89 ± 6.3^a^ |
| Height | 30 months | 93 ± 4.39^a^ | 92.5 ± 3.54^a^ | 93 ± 8.28^a^ |
| Height | 36 months | 100 ± 3^a^ | 97.3 ± 6.61^a^ | 98 ± 3.66^a^ |
| BMI | 05 days | 13.8 ± 1.9^a^ | 13.4 ± 2.07^a^ | 14.1 ± 2.47^a^ |
| BMI | 42 days | 15.2 ± 1^a^ | 15 ± 1.48^a^ | 15.5 ± 3.51^a^ |
| BMI | 03 months | 17.8 ± 1.75^a^ | 17.7 ± 3.47^a^ | 17.8 ± 1.69^a^ |
| BMI | 06 months | 17.9 ± 1.78^a^ | 18.3 ± 1.42^a^ | 18.4 ± 3.3^a^ |
| BMI | 18 months | 16.6 ± 2.09^a^ | 20.7 ± 4.45^a^ | 16.6 ± 2.92^a^ |
| BMI | 24 months | 15.5 ± 1.69^a^ | 17.2 ± 1.75^a^ | 16.9 ± 4.77^a^ |
| BMI | 30 months | 15.6 ± 2.45^a^ | 14.7 ± 0.77^a^ | 15.7 ± 1.73^a^ |
| BMI | 36 months | 16.1 ± 1.4^a^ | 16 ± 2.4^a^ | 16.3 ± 1.38^a^ |

Note: Row values with different superscript letters are significantly different. BMI, body mass index; CC, control group (pregnant women with a BMI ≥24 kg/m^2^); NC, normal weight group (pregnant women with a BMI <23.9 kg/m^2^); YC, yoghurt intervention group (pregnant women with a BMI ≥24 kg/m^2^).
