# Supplementary material for: Maternal overweight/obesity and yoghurt supplementation from early pregnancy to postpartum augments infant gut microbiota
Source: Front Nutr. 2026 Feb 26;13:1733803. doi: 10.3389/fnut.2026.1733803 (PMC12979164; doi:10.3389/fnut.2026.1733803)
Supplement: Supplementary file 4 [file Image_1.pdf]

## Supplementary Figure

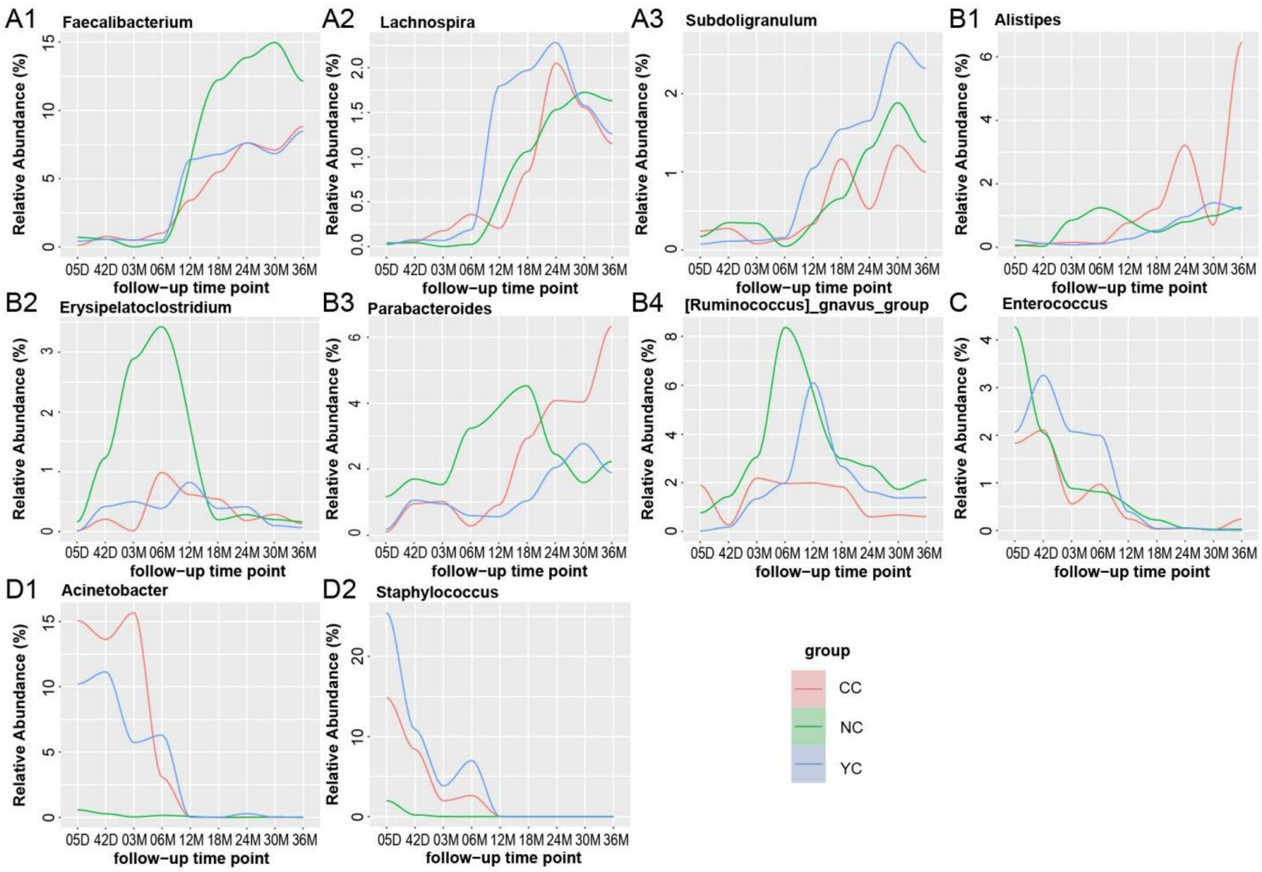


**Supplementary Figure 1.** Changes in the relative abundance of gut microbiota in the three groups of infants. **(A)** Changes in the abundance of gut microbiota that follow pattern 1: initially stable, followed by an increase over time in the three groups. A1, A2, and A3 represent *Faecalibacterium*, *Lachnospira*, and *Subdoligranulum*, respectively. **(B)** Changes in the abundance of gut microbiota that follow pattern 2: increasing and then decreasing over time in the three groups. B1–B4 represent *Alistipes*, *Erysipelatoclostridium*, *Parabacteroides*, and [*Ruminococcus*]_*gnavus*_group, respectively. **(C)** Changes in the abundance of *Enterococcus* in the gut microbiota that follow pattern 3: decreasing over time in the three groups. **(D)** Changes in the abundance of gut microbiota that follow pattern 4: stable over time in the three groups. D1 and D2 represent *Acinetobacter* and *Staphylococcus*, respectively.
